# Fusion of MALDI Spectrometric Imaging and Raman Spectroscopic Data for the Analysis of Biological Samples

**DOI:** 10.3389/fchem.2018.00257

**Published:** 2018-07-16

**Authors:** Oleg Ryabchykov, Juergen Popp, Thomas Bocklitz

**Affiliations:** ^1^Spectroscopy and Imaging Research Department, Leibniz Institute of Photonic Technology, Member of Leibniz Health Technology, Jena, Germany; ^2^Institute of Physical Chemistry and Abbe Center of Photonics, Friedrich Schiller University Jena, Jena, Germany

**Keywords:** MALDI-TOF, Raman imaging, data combination, data fusion, normalization, PCA

## Abstract

Despite of a large number of imaging techniques for the characterization of biological samples, no universal one has been reported yet. In this work, a data fusion approach was investigated for combining Raman spectroscopic data with matrix-assisted laser desorption/ionization (MALDI) mass spectrometric data. It betters the image analysis of biological samples because Raman and MALDI information can be complementary to each other. While MALDI spectrometry yields detailed information regarding the lipid content, Raman spectroscopy provides valuable information about the overall chemical composition of the sample. The combination of Raman spectroscopic and MALDI spectrometric imaging data helps distinguishing different regions within the sample with a higher precision than would be possible by using either technique. We demonstrate that a data weighting step within the data fusion is necessary to reveal additional spectral features. The selected weighting approach was evaluated by examining the proportions of variance within the data explained by the first principal components of a principal component analysis (PCA) and visualizing the PCA results for each data type and combined data. In summary, the presented data fusion approach provides a concrete guideline on how to combine Raman spectroscopic and MALDI spectrometric imaging data for biological analysis.

## Introduction

Different analytical methods could be utilized for biomedical analysis (e.g., cells, and tissues, etc.) to highlight a certain aspect of the sample e.g., morphological microstructure, distribution of electronic chromophores, molecule classes, or special proteins. Among the label-free imaging approaches, matrix-assisted laser desorption/ionization (MALDI) spectrometry, and Raman microscopy are certainly among the most powerful imaging techniques for the investigation of biomedical samples. Raman spectroscopy is a non-destructive spectroscopic method, which provides complex molecular information about the general chemical composition of the sample with a rather high spatial resolution (Abbe limit) to highlight subcellular features (Kong et al., 2015). The drawback of Raman imaging lies in its weak scattering efficiency that makes sampling time rather long for large area imaging. Raman spectroscopic imaging has demonstrated its potential for biomedical diagnosis in numerous cancer-related studies (Tolstik et al., 2014), biological material analysis (Butler et al., 2016), cell characterization studies (Ramoji et al., 2012), and many other biomedical applications (Matousek and Stone, 2013; Ember et al., 2017).

On the other side, MALDI mass spectrometry provides information on specific substances, such as lipids or proteins (Fitzgerald et al., 1993). MALDI is a soft ionization technique utilized for mass-spectrometric imaging (Gessel et al., 2014) to determine large organic molecules and biomolecules undetected by conventional ionization techniques. This technique was employed in clinical parasitology (Singhal et al., 2016), microbial identification (Urwyler and Glaubitz, 2016), and cancer tissue investigation (Hinsch et al., 2017).

Raman spectroscopic and MALDI mass spectrometric imaging both offer a high molecular sensitivity. Moreover, Raman spectroscopy has been sequentially applied together with different mass spectrometric techniques to address a variety of biological tasks such as characterization of succinylated collagen (Kumar et al., 2011), investigation of microbial cells (Wagner, 2009), identification of fungal strains (Verwer et al., 2014) and characterization of lipid extracts from brain tissue (Köhler et al., 2009). In all the aforementioned studies, the Raman and mass spectrometric data are analyzed separately, and then summarized or compared to each other (Masyuko et al., 2014; Bocklitz et al., 2015; Muhamadali et al., 2016). To significantly increase the information content, Raman spectroscopic and MALDI mass spectrometric imaging data have to be co-registered (Bocklitz et al., 2013) followed by a high-level (distributed) data fusion. It means that each data type is analyzed separately to obtain the respective scores, which are then fused together. Alternatively, spectroscopic imaging can be used for mapping an area that is suitable for further investigation by means of MALDI spectrometric imaging (Fagerer et al., 2013) or a certain mass peak is used to define an area, from which the Raman spectra are analyzed (Bocklitz et al., 2013). Such a hierarchical pipeline corresponds to a decentralized data fusion approach.

In the present work, we introduced an analytical method to perform a low-level (centralized) fusion of Raman and MALDI imaging data. Because the experimental implementation of correlated imaging is challenging in many aspects (Masyuko et al., 2013), we utilized a computational approach to combine imaging data obtained by MALDI spectrometry and Raman spectroscopy. The correlation of Raman spectroscopy with mass spectrometric imaging techniques such as MALDI (Ahlf et al., 2014) or secondary ion mass spectrometry (SIMS) (Lanni et al., 2014) have proved its usefulness for biological applications. Moreover, a combination of MALDI imaging data with optical microscopy could attenuate instrumental effects (Van De Plas et al., 2015), and a joint analysis of vibrational and MALDI mass spectra could provide valuable information on brain tissue (Van De Plas et al., 2015; Lasch and Noda, 2017). Nevertheless, even if Raman and MALDI spectra are obtained by correlated imaging, each type of spectra shows its own specific features and should be preprocessed separately. Because the measurement techniques are based on different physical effects, the difference in data dimensionality and dynamic range can affect the contribution of each datatype in the analysis. Therefore, a weighting coefficient that balances the influence of Raman spectroscopic and MALDI spectrometric data in the data fusion center is required.

## Materials and methods

### Experimental details

We demonstrated the data fusion on an example dataset of MALDI spectrometric and Raman spectroscopic scans obtained from the same mouse brain sample (*Mus musculus*) of 10 μm cryosection. The sample was cut on a cryostat, and then dried on a precooled conductive ITO-coated glass slide. Subsequently, Raman spectra were obtained using a confocal Raman microscope CRM-alpha300R (WITec, Ulm, Germany) and excited with a 633 nm HeNe laser (Melles Griot). The laser irradiation was adjusted in order to have about 10 mW power. The laser was coupled through an optical fiber into a Zeiss microscope. A spectral map was obtained by a raster scan with a 25 μm grid with a dwell time of 2 s and a pre-bleaching time of 1 s.

After the Raman scan, MALDI mass spectrometric imaging was performed with a common matrix alpha-cyano 4-hydroxy cinnamic acid (5 mg/mL) in 50% acetonitrile and 0.2% trifluoracetic acid. The ImagePrep station (Bruker Daltonics) was used to prepare and apply the matrix on the sample. The MALDI-time-of-flight (MALDI-TOF) spectrometric map was obtained on a Ultraflex III MALDI-TOF/TOF mass spectrometer (Bruker Daltonics, Bremen, Germany). A “smartbeam” laser (λ = 355 nm, repetition rate 200 Hz) was used. The spectrometer was calibrated with an external standard, a peptide calibration mixture (Bruker Daltonics). The measurements were performed in the positive reflectron mode with 500 shots per spectrum and spatial resolution of 75 μm.

Further experimental details for both data types and an example of a hierarchical data fusion implementation can be found in the report by Bocklitz et al. (2013). Nevertheless, in the context of a further discussion, it is important to highlight that in MALDI mass spectrometric imaging a matrix suitable for the analysis of the lipid content was applied.

### Preprocessing of Raman spectroscopic data

**Figure 1:**
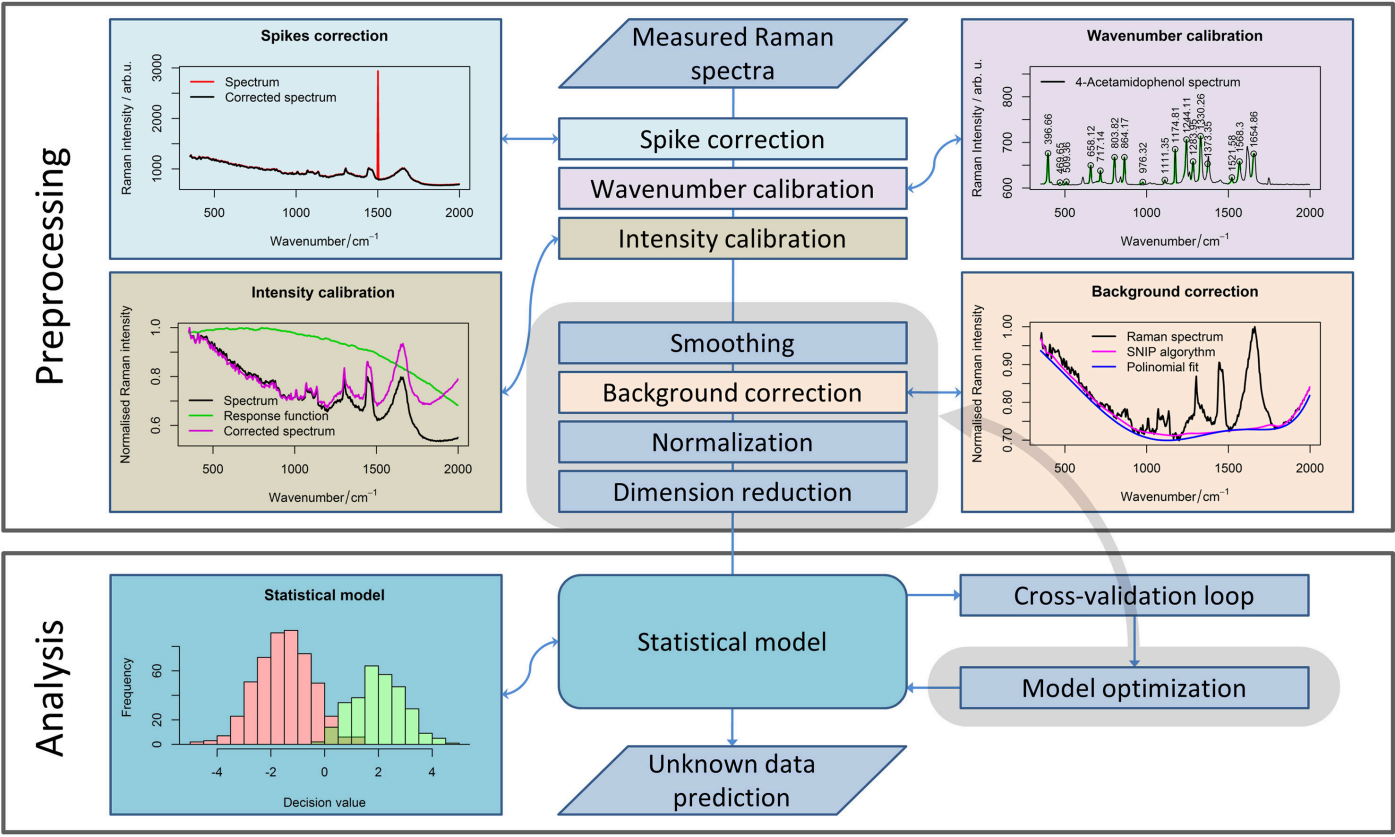
A general pipeline for Raman data preprocessing. The workflow shows the main steps of the preprocessing routine necessary for robust Raman spectral analysis. Although some steps should be defined while planning the experiment, subsequent preprocessing methods (highlighted in gray area) and their parameters can be optimized for extracting the required information from the data.

The influence of corrupting effects (e.g., cosmic spikes, fluorescence) on Raman spectra cannot be avoided completely. Thus, the development of complex preprocessing routines (Bocklitz et al., 2011) is required. To allow further analysis of the Raman spectra obtained with different calibrations, all spectra need to be interpolated to the same wavenumber axis (Dörfer et al., 2011). Moreover, keeping all the spectra in a single data matrix simplifies a further processing routine, so it is advantageous to perform the calibration as one of the first steps of the preprocessing workflow (Figure 1). Besides the wavenumber calibration, intensity calibration should be performed for the comparison of the measurements obtained with different devices or in the case where some changes in the measurement device have occurred (Dörfer et al., 2011).

The calibration is always needed for a reliable analysis, especially if the measurements were performed over a large time period, or settings of the device were changed between the measurements. In contrast, the following step within the preprocessing workflow (i.e., noise removal) is an optional step. However, among smoothing methods, only the running median with a relatively large window is applicable for cosmic ray noise removal. Unfortunately, filtering with a large window may corrupt the Raman bands themselves. Alternatively, 2–3 spectra per point can be acquired to eliminate the spikes that are not present in each spectrum. Nevertheless, this approach increases the measurement time dramatically. Therefore, this approach is not suitable for Raman imaging when a large number of spectra are recorded. Thus, specialized spike correction approaches like wavelet transform (Ehrentreich and Summchen, 2001), correlation methods (Cappel et al., 2010), calculation of the Laplacian of the spectral data matrix (Schulze and Turner, 2014; Ryabchykov et al., 2016), or a difference between the original and a smoothed spectrum (Zhang and Henson, 2007) must be used for spike removal.

The next step in the preprocessing workflow for Raman spectra is fluorescence background removal. In this work, the sensitive nonlinear iterative peak (SNIP) clipping algorithm (Ryan et al., 1988) was used for baseline estimation. The SNIP algorithm can be utilized for background estimation for a number of spectral measurements, like X-ray and mass spectra.

After baseline correction, the Raman spectra must be normalized (Afseth et al., 2006) to complete the basic preprocessing. There are several normalization approaches (e.g., vector normalization, normalization to integrated spectral intensity, or a single peak intensity value) that enhance the stability of the spectral data. In this work, we used vector normalization and *l*_1_-normalization (Horn and Johnson, 1990) for Raman spectra. The difference between normalization to integrated spectral intensity and *l*_1_-normalization is that the latter utilized absolute intensity values. As a result, the difference between both normalization approaches becomes more significant when negative values appear in the baseline corrected spectra due to noise or baseline correction artifacts.

### Preprocessing of MALDI spectrometric data

**Figure 2:**
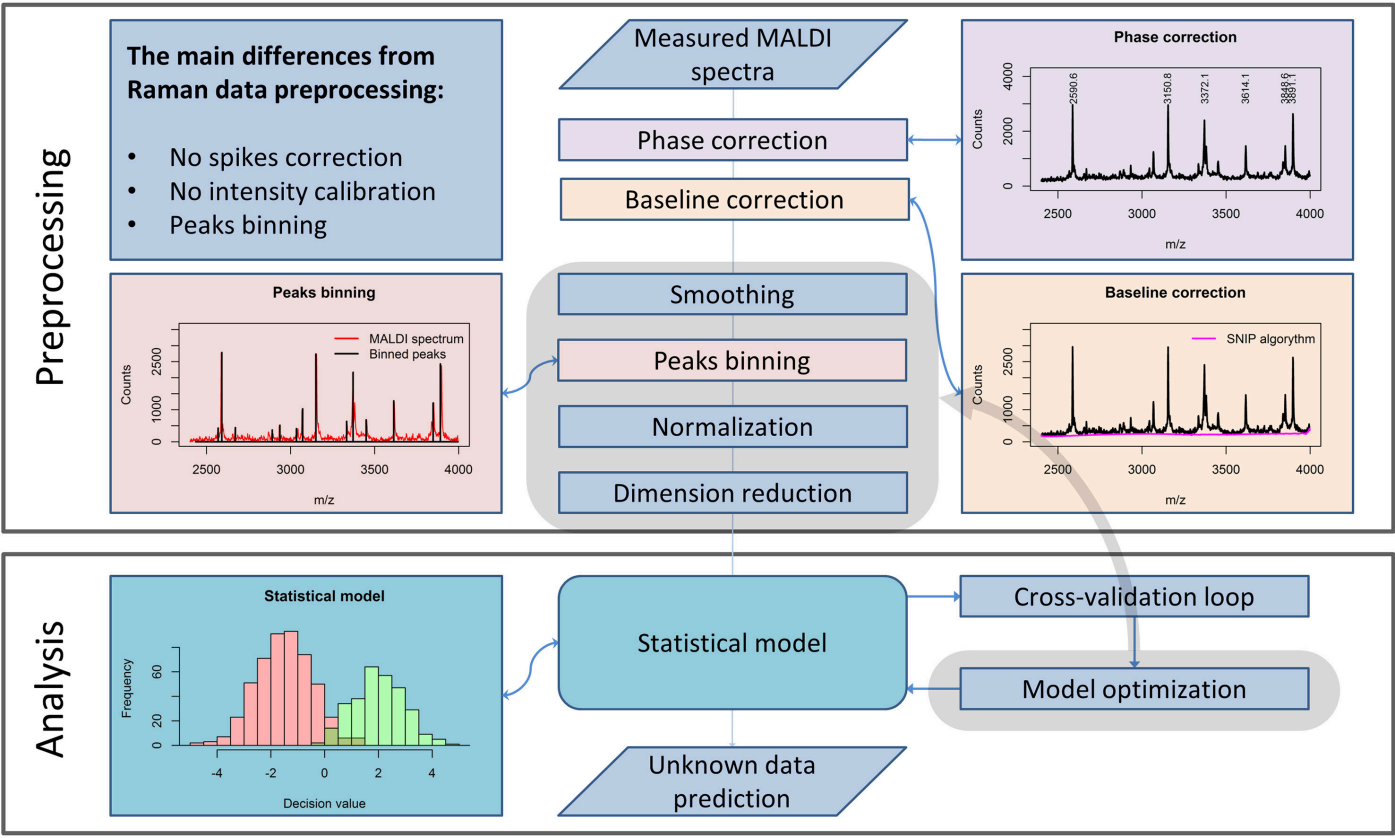
A general pipeline for MALDI data preprocessing. The workflow shows the main steps of the preprocessing routine necessary for robust MALDI spectral data analysis and the main differences as compared to the Raman data preprocessing routine, described in Figure 1.

Although the measurement techniques themselves differ dramatically for Raman and MALDI mass spectroscopic imaging data, the preprocessing of these data has a lot in common. The m/z values are set according to an internal calibration and may “float” slightly from one measurement to another. Therefore, a phase correction along the m/z axis must be performed within the preprocessing workflow (Figure 2) to ensure that the spectra obtained in different measurements are comparable. For this purpose, it is advisable to use the stable intense peaks within the phase correction routine (Gu et al., 2006).

From a theoretical point of view, MALDI spectra should not feature a spectral background. Nevertheless, in measured MALDI spectra a background is present. In literature, a background present in MALDI mass spectra is also known as “chemical noise background” (Krutchinsky and Chait, 2002). This type of noise results from matrix impurities and unstable ion clusters created during the sample scanning.

Similarly to Raman spectral preprocessing, the SNIP algorithm (Ryan et al., 1988) can be used to eliminate the background from mass spectra. Another complication in the analysis of MALDI spectra results from the fact that even after the phase correction, peak positions vary insignificantly among different spectra. An interpolation procedure, which is applied in Raman data preprocessing, would corrupt the sharp peaks found in MALDI spectra and is therefore not applied. To enable a direct comparison of the spectra, a binning procedure is applied. This procedure is based on the equalization of the m/z-values of peak positions within a certain range. Since the average peak width along the m/z axis increases with increased mass, the binning range is set with a so-called tolerance relative to the mass values. In contrast to Raman spectroscopy, intensity calibration for MALDI mass spectrometric imaging is not required. Nevertheless, normalization may be applied. Various types of normalization are used for MALDI mass spectroscopic imaging data: total ion count (TIC), vector norm (RMS), median, square root, logarithmic, and normalization to a noise level. In contrast to the Raman spectral data, MALDI mass spectra do not feature negative values. Thus, TIC normalization and normalization to *l*_1_-norm, which is a sum of absolute values, are equal for MALDI spectra. If the significance level of the data is high, the normalization may be not necessary for the subsequent analysis.

### Computational details

For MALDI data acquisition and calibration, a flexImaging software version 3.0 (Bruker Daltonics) was used. The data processing was also performed in R (R Core Team, 2017) using packages akima (Gebhardt)^1^, Peaks (Morhac)^2^, readBrukerFlexData (Gibb)^3^, rsvd (Erichson)^4^, spatstat (Baddeley and Turner, 2005), and Spikes (Ryabchykov et al., 2016).

Prior to the data preprocessing and data fusion, the MALDI and Raman spectra were interpolated to the same (spatial) grid by utilizing a co-registration framework. Based on the false color images of Raman spectroscopic and MALDI spectrometric scans, 6 points clearly representing the same positions on every scan were manually selected. The coordinates of the Raman spectroscopic map were then transformed to the coordinate system of the MALDI mass spectrometric map. Subsequently, the Raman spectra were interpolated to the grid of the MALDI mass spectral map. To perform this interpolation, every point within the Raman grid was assigned to the nearest point within the MALDI grid. After that, the average of the Raman spectra, assigned to the same point within the MALDI grid, was calculated. Two spectral maps were thus obtained and aligned in a point-wise manner.

After the alignment, the Raman spectroscopic and MALDI mass spectrometric imaging data were preprocessed. During the preprocessing, the wavenumber calibration of the Raman spectra and the phase correction of MALDI spectra were performed. The MALDI mass spectrometric imaging data were subsequently subjected to noise removal, background correction, and TIC normalization. The Raman spectra were corrected for fluorescence background and vector normalized. The SNIP algorithm was used for background estimation in both cases.

After the preprocessing, Raman and MALDI mass spectral data differed in their dimensionality and in dynamic range. Data with different dynamic ranges would contribute unequally in a further analysis and consequently the spectral matrices have to be additionally weighed before performing the PCA. The weighting coefficient was selected as a ratio between the *l*_1_-norms of the matrices, which are sums over the absolute values in the matrix. After the weighting, the data were combined in a single matrix and analyzed with a PCA. To illustrate the benefit of data fusion and weighting, we also analyzed the un-weighted data in a combined manner and each data type separately. We also investigated the case, where the same normalization approach was applied to both data types and no additional weighting is required. When the Raman spectra were normalized to the total spectral intensity, which is equivalent to TIC normalization of mass spectra, the data matrices had equal *l*_1_-norms.

## Results and discussion

**Figure 3:**
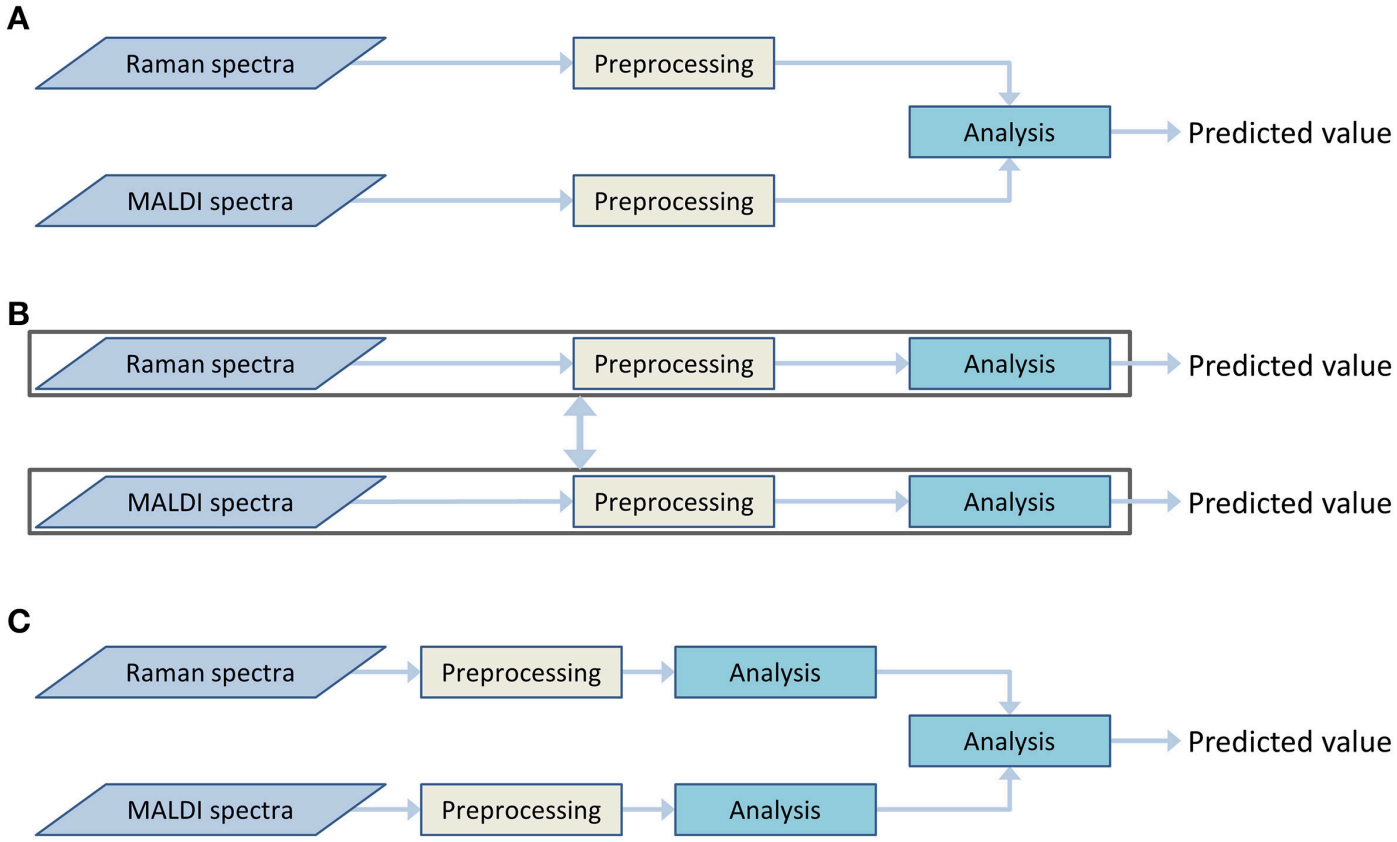
Various data fusion architectures: centralized **(A)**, decentralized **(B)**, and distributed **(C)** architectures.

Both Raman spectroscopic and MALDI mass spectrometric imaging data provide different insights into the chemical composition of the sample. Information on a broad range of molecules can be obtained from the Raman spectra. This information can be complemented by detailed information on lipid content, obtained from the MALDI data. To utilize both types of information together, a data fusion must be applied. This data fusion may be performed during different stages of the analysis workflow. Therefore, the architecture of the data processing workflow is dependent on the selected data fusion approach. These approaches can be divided into the following types (Castanedo, 2013):

- Centralized architecture (Figure 3A). The preprocessed data from different sources are combined in the data fusion center and are analyzed together.
- Decentralized architecture (Figure 3B). This scheme does not have a single data fusion center. The processing workflows are interacting at different processing stages. This architecture may provide multiple outputs or be represented as a hierarchical structure.
- Distributed architecture (Figure 3C). Each data type is preprocessed and analyzed separately. Subsequently, the output values are evaluated and combined to obtain a single result.

The decentralized and distributed architecture already showed their effectiveness for biomedical investigations (Bocklitz et al., 2013; Ahlf et al., 2014). The current work focuses on the centralized data fusion approach, also called low-level data fusion. In contrast to decentralized and distributed architectures, the centralized architecture shows a simpler workflow (Figure 3A). The data are combined in early steps of the analysis, directly after the preprocessing and even before the dimension reduction. At the data fusion center, where the different types of data are combined, an additional normalization or scaling of the data may be required to weight the influence of the different data types on the global model. The need for this weighting step arises from the differences in the data dimensionality, measurement units and dynamic ranges of the different measurement techniques. It is worth mentioning that the weighting is not a major issue in high-level data fusion approaches, which usually deal with standardized low-dimensional outputs of preliminary analysis in the data fusion center. However, a low-level data fusion (such as the applied centralized data fusion model) deals directly with preprocessed spectra of different types. Thus, the data scaling may dramatically influence extraction efficiency of the features.

To investigate the impact of data weighting, we searched for a marker that would allow an objective comparison of different data fusion and normalization approaches. This weighting scheme is designed for biological samples (i.e., a complex chemical composition), of which a large number of independent features have to be identified for appropriate description. By applying a PCA for dimension reduction, a large portion of the data variance is expected to be spread among multiple principal components (PCs) and the optimal approach should correspond to the slowest raise of the cumulative proportion of variance with a number of PCs.

**Figure 4:**
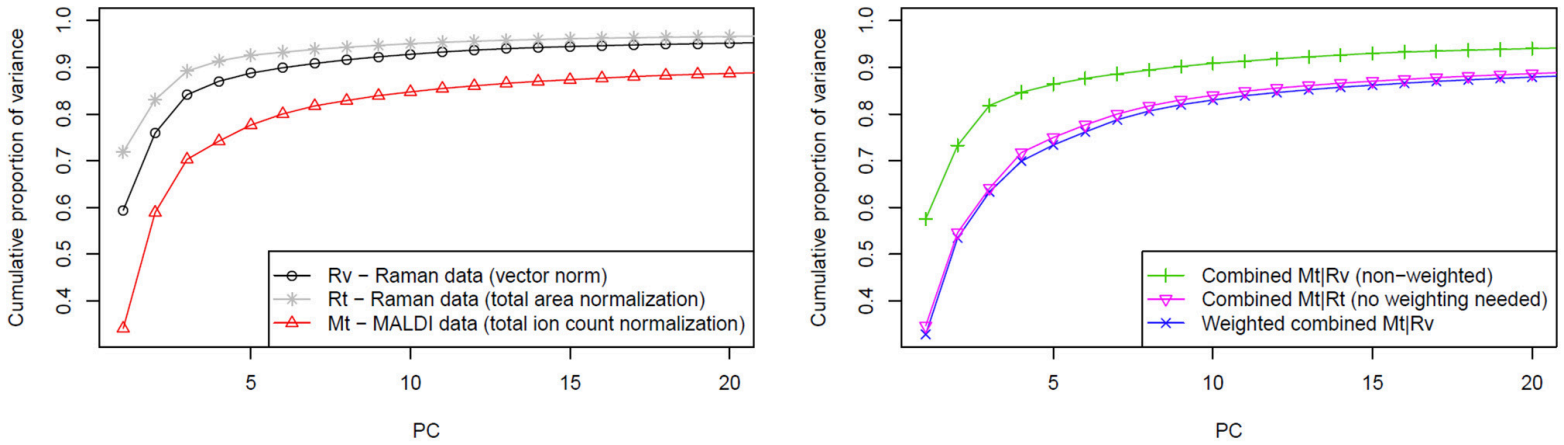
Influence of weighting in the data fusion center on the PCA. The left side of the figure shows cumulative proportion of data variance explained by first 20 PCs for Raman data (normalized in two different ways) and for MALDI data. On the right side of the figure, a slower raise of cumulative proportion of weighted data variance in comparison to the non-weighted case is shown. This trend reflects that more independent features can be extracted from the data by applying weighting prior to the data fusion. As it is also shown in the plot on the right side, a similar effect can be reached by applying the same type of normalization for both data types.

The variances of the data explained by PCA are shown in the Figure 4 where the normalization and fusion approaches (described in section Computational Details) are shown. Unfortunately, a direct comparison between cumulative proportions of variance obtained from Raman and MALDI mass spectral data, and their combined data is not suitable due to the different number of variables. However, different trends in the observed variance by the PCs in data with the same dimensionality can be interpreted. The left side of Figure 4 shows that the variance of vector normalized Raman data is spread among a larger number of PCs than that of the total area normalized Raman data. This finding indicates that the vector normalization allows extracting a larger number of significant features from Raman data. Because the Raman spectra were vector normalized and the MALDI spectra were TIC normalized, the Raman data contribute more to the overall data variance than the MALDI data. Consequently, the PCA will focus on the variations in the Raman data and the variations in the MALDI data will have only a small influence. Alternatively, two datasets can be balanced by normalizing spectra of both types to their *l*_1_-norms. By definition, this norm is a sum of absolute values. It takes dimensionality and scaling of the data into account, so no additional weighting is required. TIC normalization performed on MALDI data is already equal to *l*_1_-normalization because there are no negative values present in the mass spectra. The right side of Figure 4 clearly shows that there is a marked difference between the approach not taking the data scaling into account and the approaches based on weighting or identical normalization. However, no significant benefit was observed when comparing the weighting to identical normalization approach.

**Figure 5:**
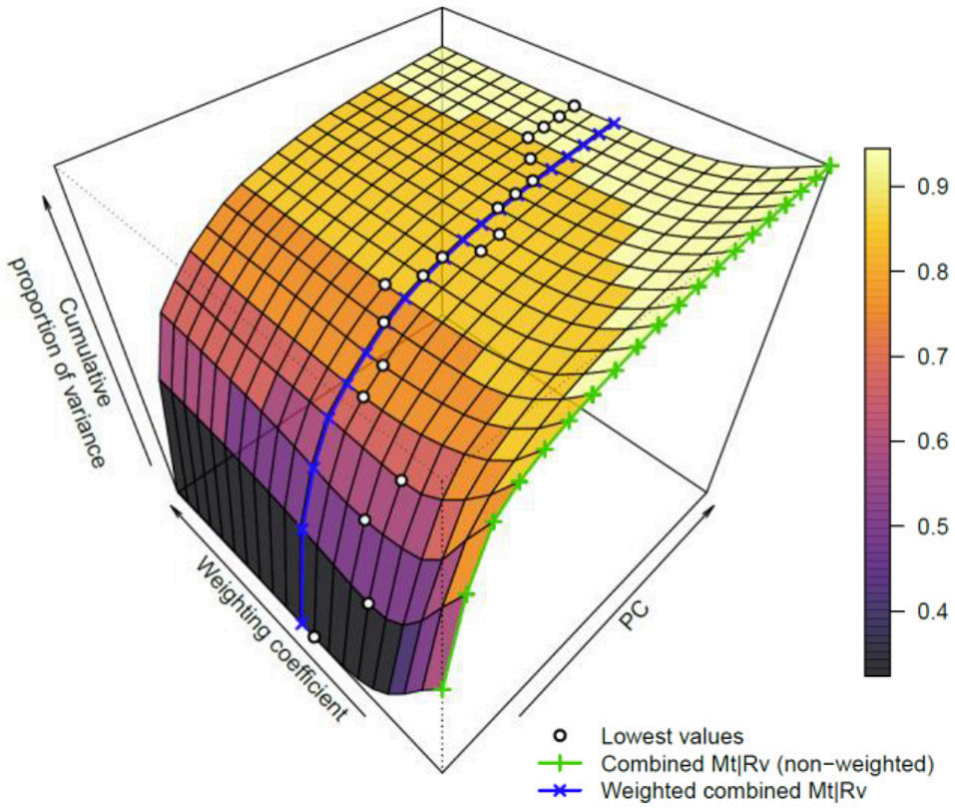
Dependence of the variance explained by PCA using the weighting scheme. The surface plot covers the first 20 PCs and weighting coefficients between 1 and 20. The cumulative proportions of variance for the weighted and non-weighted cases are shown as blue and green lines, respectively (please refer to the online version for colors). Furthermore, the lowest variance is highlighted for each number of PCs with a dot. These dots represent an optimal unmixing for the related number of PCs. Although this optimum changes with respect to PC numbers, the used weighting coefficient based on *l*_1_-norms clearly lies near the minimum of cumulative proportion of variance for a given number of PCs.

To further investigate the influence of weighting on data fusion, the weighting coefficient was varied in a range from 1 to 20 and a PCA utilized for every case. The extracted curves of the cumulative proportion of the variance were organized as a surface plot (Figure 5). To make the interpretation easier, the curves, which correspond to the data combination without weighting and with weighting based on the ratio of *l*_1_-norms, are additionally highlighted in Figure 5. Although no single weighting coefficient is globally the best, the proposed weighting coefficient lies close to the area where the data variance is spread between multiple PCs. Thus, fusing data in this manner enables the PCA to extract a larger number of reliable features.

**Figure 6:**
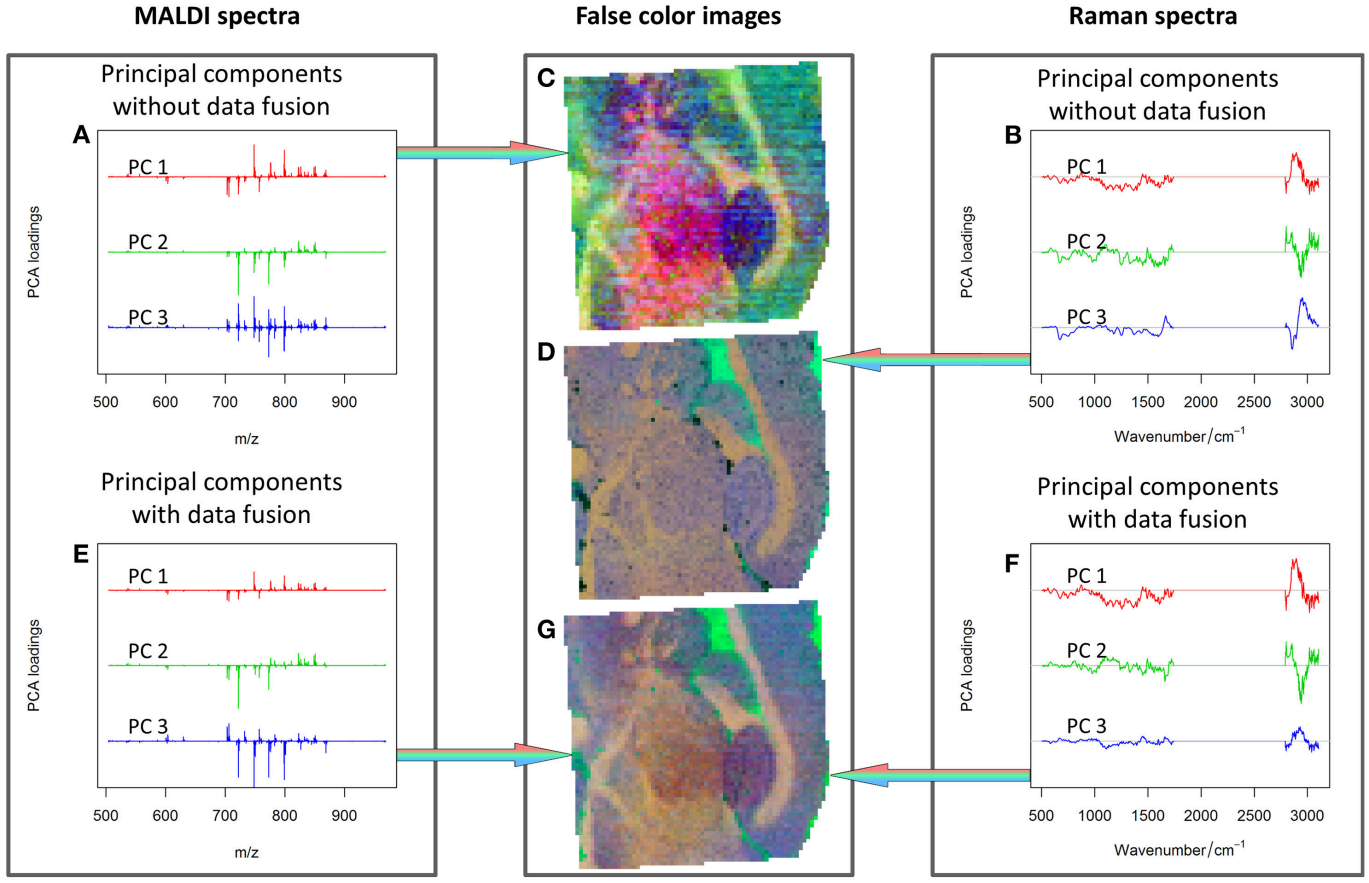
PCA analysis: first three PCs calculated for MALDI spectra **(A)**, Raman spectra **(B)**, combined Raman-MALDI data **(E,F)** and their false-color score composites **(C,D,G)**. Red, green, and blue colors indicate the first, second and third PCs, respectively. Separate plots for the loadings and false color images can be found as Supplementary Material. The PCs composite image of the combined data **(G)** shows a smoother appearance, and the loadings after data fusion **(E,F)** are easier to interpret. See text for further details.

Although an optimal data fusion has been achieved as above-mentioned, a direct comparison of cumulative proportions of variance explained by the PCA for data with different dimensionalities may be misleading. Hence, the results obtained from the combined approach and separated data analysis (Figure 6) were checked by means of inspecting the PCA loadings and scores. The first three PCs were visualized separately for the MALDI spectrometric imaging data (Figures 6A,C), Raman spectroscopic imaging data (Figures 6B,D), and their combination (Figures 6E–G).

**Figure 7:**
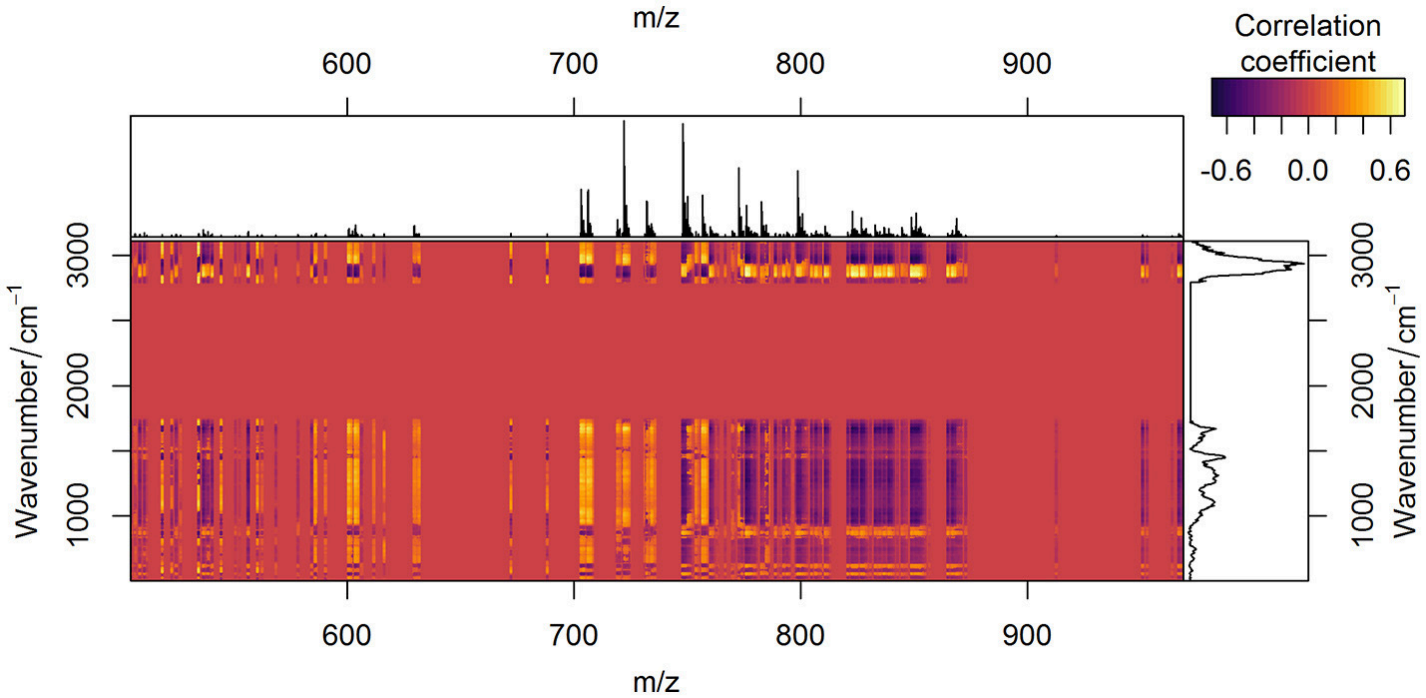
Correlation between Raman spectroscopic and MALDI mass spectrometric data. Correlation of two data types after being preprocessed is depicted in yellow (positive values), red (zero), and violet (negative values) colors. Average preprocessed MALDI spectrum (on the top of the figure) and Raman spectrum (on the right side of the figure) are plotted for easier interpretation.

The comparison of the PCA scores in Figure 6 shows that the image of the MALDI-Raman combination (Figure 6G) depicts clearer spatial features of the sample (compared to Figures 6C,D). The corresponding false-color score composite (Figure 6G) is less noisy, and looks subjectively better than the images obtained separately from the MALDI mass spectrometric (Figure 6C) and Raman spectroscopic data (Figure 6D). Moreover, the loading vector of the third PC of the MALDI spectra (shown in blue color in Figure 6A) has positive and negative values related to isotopes of the same molecules. It means that it represents mostly noise and variations in the signal to noise ratio. On the other hand, the MALDI part of the loadings of the third PC in the combined analysis (shown in blue color in Figure 6E) reflects a joint behavior for the isotopes of the same ions. Moreover, the Raman part of this PC contains the peaks associated with lipids (Notingher and Hench, 2006), namely the C = C stretching region (1,655–1,680 cm^−1^), and CH deformation band (1,420–1,480 cm^−1^). Although these two peaks may also be associated with Amide I and CH deformations of proteins, there is a decrease in the protein-associated range (Notingher and Hench, 2006) in the wavenumber region 1,128–1,284 cm^−1^. Furthermore, there are notable changes in the CH-stretching region (2,800–3,100 cm^−1^). Thus, the third PC of the combined data represents the actual diversity in the lipid composition of the sample. The relationship of the CH stretching region of the Raman spectra to the changes in the lipid content can also be observed by a high correlation of the Raman spectral region with MALDI mass spectra (Figure 7).

Since both data types simultaneously reflect variations in lipid content, the specific changes in the correlation profiles (Figure 7) of the Raman and MALDI data are observed in the areas related to lipid bands in Raman spectra. Besides the contributions of lipids, which are found in the third PC, the fingerprint region of Raman spectra contains numerous peaks related to proteins and DNA. These Raman bands correlate with MALDI peaks both positively and negatively (Figure 7). The correlation of a certain MALDI peak with the Raman data shows a similar structure, but with an opposite sign. This sign change reflects changes in the contribution of specific lipids with respect to the overall increase of lipid content in the sample.

One of the non-lipid compounds, which feature strong Raman bands, is phenylalanine. Its symmetrical ring breathing mode and C-H in-plane mode are visible in the first two PCs at 1,004 and 1,030 cm^−1^. Another peak related to phenylalanine can be found in the first two PCs at 1,104 cm^−1^ (Movasaghi et al., 2007). Aside of that, the first PC contains contributions of tryptophan at 760 cm^−1^ (Bonifacio et al., 2010). The protein backbone C-C_α_ stretching of collagen is present in the second PC at 936 cm^−1^ and the ν(C–C) protein backbone is located in the first two PCs at 816 cm^−1^ (Bonifacio et al., 2010). Also, prominent collagen-associated bands like Amide I and Amide III can be seen in the first PC at 1,655–1,680 and 1,220–1,284 cm^−1^, respectively (Krafft et al., 2005; Notingher and Hench, 2006). Moreover, the peak at 1,647 cm^−1^ is associated with the random coil structure of proteins in general (Movasaghi et al., 2007). This peak is also present in the first two PCs.

The main contribution to the first PC is the ratio between the fingerprint region of Raman spectra and C-H stretching region. On the other side, the fingerprint region of the second PC contains both positive and negative peaks, reflecting the changes in protein content. Along with the protein content, valuable information about DNA is obtained from the first two PCs of the Raman spectra. The peak at 1,180 cm^−1^ represents cytosine and guanine. Another DNA peak is located at 1,263 cm^−1^ and represents adenine and thymine (Movasaghi et al., 2007). All Raman spectral features provide a complex overview of the chemical composition of the mouse brain section. The MALDI data, on the other hand, extends the overview of the distribution of biomolecules based on Raman spectroscopy with detailed information about the lipid content composition.

## Conclusion

In this paper, a data fusion scheme was investigated to analyze Raman spectroscopic and MALDI mass spectrometric imaging data together. We described the most significant corrupting effects influencing the analysis of Raman spectroscopic and MALDI mass spectrometric imaging data. The preprocessing workflows were shown for the suppression of these corrupting effects by means of calibration, noise reduction, background correction, and normalization for both data types. After the pretreatment steps, the importance of data weighting prior to data fusion is highlighted, especially when the data are obtained from different sources and have different scales and dimensionalities. As there is no universal way of balancing the influence of data types on the analysis, optimization, and validation of weighting approaches should be done according to the specific data. In order to allow a judgment of the quality of a weighting, we proposed an approach that allows estimating the goodness of data weighting. This approach is based on analyzing proportions of data variance explained by PCs and we applied this approach by examining the cumulative variance. It was shown that the weighting, based on the ratio of *l*_1_-norms of the data matrices, allows optimal unmixing of the example data set into features. Besides the comparison of different weighting schemes, the proposed method can be used for the comparison of normalization approaches. It was found that vector normalization allows better unmixing of the example Raman data as compared to the normalization to the integrated spectral intensity (*l*_1_-norm). Besides the establishment of a weighting approach, we discovered that a nearly optimal result compared to the weighting is achieved if the spectra of both types are normalized to the same norm. We could demonstrate this by normalizing both types of spectra of an example dataset to the same norm. This was the *l*_1_-norm in our example. However, it is important to keep in mind that this method of comparing the cumulative proportions of variance should be used only when a researcher is interested in maximizing the number of extracted independent features.

The revealing of additional meaningful features by means of optimal data fusion was demonstrated for the combination of Raman spectroscopic and MALDI mass spectrometric imaging data. We showed this by comparing the third PC extracted from each type of data separately and from the combined data. The MALDI-related part of the third combined component showed a clearer interpretation in comparison to the third loading obtained from the MALDI data alone. Moreover, the Raman-related part of the combined component reflected variations in lipid to protein ratio. This PC depicts a decrease in a protein-associated range that occurs along with an increase of bands related to the CH deformation and C = C stretching in lipids, which can be found in the regions 1,128–1,284, 1,420–1,480, and 1,655–1,680 cm^−1^, respectively. Therefore, changes in the lipid to protein ratio and changes in lipid content itself can be observed simultaneously through the data fusion of Raman spectroscopic and MALDI mass spectrometric imaging data.

Finally, the advantage of the combined analysis was illustrated by a comparison of the PCA results visualized as false-color RGB images. These images were obtained separately for the preprocessed Raman and MALDI imaging data and for the combined data. Visual investigation of the images showed that the combined approach provides a sharper image with less noise contributions. This allows the conclusion that the data fusion increases reliability not only for the spectral but also for the spatial features present in the data.

## Ethics statement

This research is based on already published data provided to the authors by Bocklitz et al. (2013). For this reason, an ethics approval was not required as per institutional and national guidelines.

## Author contributions

TB and JP initiated the study, supervised the study and discussed the results. OR performed the analysis including the development of the R scripts. TB performed the pre-study including the co-registration step. OR, JP, and TB wrote the manuscript.

### Conflict of interest statement

The authors declare that the research was conducted in the absence of any commercial or financial relationships that could be construed as a potential conflict of interest.

## Footnotes

^1^Gebhardt, H. A. “akima: Interpolation of Irregularly and Regularly Spaced Data.”

^2^Morhac, M. “Peaks: Peaks.”

^3^Gibb, S. “readBrukerFlexData: Reads Mass Spectrometry Data in Bruker ^*^flex Format.”

^4^Erichson, N. B. “rsvd: Randomized Singular Value Decomposition.”

## Supplementary material

Supplementary Image 1 The plots from Figure 6 provided in vector format.

## References

[B1] Afseth N. K. Segtnan V. H. Wold J. P. (2006). Raman spectra of biological samples: a study of preprocessing methods. Appl. Spectrosc. 60, 1358–1367. 10.1366/000370206779321454 17217584

[B2] Ahlf D. R. Masyuko R. N. Hummon A. B. Bohn P. W. (2014). Correlated mass spectrometry imaging and confocal Raman microscopy for studies of three-dimensional cell culture sections. Analyst 139, 4578–4585. 10.1039/C4AN00826J 25030970

[B3] Baddeley A. Turner R. (2005). spatstat: an R package for analyzing spatial point patterns. J. Stat. Softw. 12:42 10.18637/jss.v012.i06

[B4] Bocklitz T. Bräutigam K. Urbanek A. Hoffmann F. Von Eggeling F. Ernst G.. (2015). Novel workflow for combining Raman spectroscopy and MALDI-MSI for tissue based studies. Anal. Bioanal. Chem. 407, 7865–7873. 10.1007/s00216-015-8987-5 26374565

[B5] Bocklitz T. Walter A. Hartmann K. Rösch P. Popp J. (2011). How to pre-process Raman spectra for reliable and stable models? Anal. Chim. Acta 704, 47–56. 10.1016/j.aca.2011.06.043 21907020

[B6] Bocklitz T. W. Crecelius A. C. Matthäus C. Tarcea N. Von Eggeling F. Schmitt M.. (2013). Deeper understanding of biological tissue: quantitative correlation of MALDI-TOF and Raman imaging. Anal. Chem. 85, 10829–10834. 10.1021/ac402175c 24127731

[B7] Bonifacio A. Beleites C. Vittur F. Marsich E. Semeraro S. Paoletti S.. (2010). Chemical imaging of articular cartilage sections with Raman mapping, employing uni- and multi-variate methods for data analysis. Analyst 135, 3193–3204. 10.1039/c0an00459f 20967391

[B8] Butler H. J. Ashton L. Bird B. Cinque G. Curtis K. Dorney J.. (2016). Using Raman spectroscopy to characterize biological materials. Nat. Protocols 11, 664–687. 10.1038/nprot.2016.036 26963630

[B9] Cappel U. B. Bell I. M. Pickard L. K. (2010). Removing cosmic ray features from Raman map data by a refined nearest neighbor comparison method as a precursor for chemometric analysis. Appl. Spectro. 64, 195–200. 10.1366/000370210790619528 20149281

[B10] Castanedo F. (2013). A review of data fusion techniques. Sci. World J. 2013:19. 10.1155/2013/704504 24288502 PMC3826336

[B11] Dörfer T. Bocklitz T. Tarcea N. Schmitt M. Popp J. (2011). Checking and improving calibration of Raman spectra using chemometric approaches. Zeitschrift Fur Phys. Chem. 225, 753–764. 10.1524/zpch.2011.0077

[B12] Ehrentreich F. Sümmchen L. (2001). Spike removal and denoising of Raman spectra by wavelet transform methods. Anal. Chem. 73, 4364–4373. 10.1021/ac0013756 11569832

[B13] Ember K. J. I. Hoeve M. A. McAughtrie S. L. Bergholt M. S. Dwyer B. J. Stevens M. M.. (2017). Raman spectroscopy and regenerative medicine: a review. Regenerat. Med. 2:12. 10.1038/s41536-017-0014-3 29302348 PMC5665621

[B14] Fagerer S. R. Schmid T. Ibáñez A. J. Pabst M. Steinhoff R. Jefimovs K.. (2013). Analysis of single algal cells by combining mass spectrometry with Raman and fluorescence mapping. Analyst 138, 6732–6736. 10.1039/c3an01135f 24027777

[B15] Fitzgerald M. C. Parr G. R. Smith L. M. (1993). Basic matrixes for the matrix-assisted laser desorption/ionization mass spectrometry of proteins and oligonucleotides. Anal. Chem. 65, 3204–3211. 10.1021/ac00070a007 8291672

[B16] Gessel M. M. Norris J. L. Caprioli R. M. (2014). MALDI imaging mass spectrometry: Spatial molecular analysis to enable a new age of discovery. J. Prot. 107, 71–82. 10.1016/j.jprot.2014.03.021 24686089 PMC4104210

[B17] Gu M. Wang Y. Zhao X. G. Gu Z. M. (2006). Accurate mass filtering of ion chromatograms for metabolite identification using a unit mass resolution liquid chromatography/mass spectrometry system. Rapid Commun. Mass Spectrosc. 20, 764–770. 10.1002/rcm.2377 16463359

[B18] Hinsch A. Buchholz M. Odinga S. Borkowski C. Koop C. Izbicki J. R.. (2017). MALDI imaging mass spectrometry reveals multiple clinically relevant masses in colorectal cancer using large-scale tissue microarrays. J. Mass Spectro. 52, 165–173. 10.1002/jms.3916 28117928

[B19] Horn R. A. Johnson C. R. (1990). Matrix Analysis. Cambridge University Press.

[B20] Köhler M. Machill S. Salzer R. Krafft C. (2009). Characterization of lipid extracts from brain tissue and tumors using Raman spectroscopy and mass spectrometry. Anal. Bioanal. Chem. 393, 1513–1520. 10.1007/s00216-008-2592-9 19153721

[B21] Kong K. Kendall C. Stone N. Notingher I. (2015). Raman spectroscopy for medical diagnostics — From *in-vitro* biofluid assays to *in-vivo* cancer detection. Adv. Drug Deliv. Rev. 89, 121–134. 10.1016/j.addr.2015.03.009 25809988

[B22] Krafft C. Knetschke T. Funk R. H. W. Salzer R. (2005). Identification of organelles and vesicles in single cells by Raman microspectroscopic mapping. Vibrat. Spectrosc. 38, 85–93. 10.1016/j.vibspec.2005.02.008

[B23] Krutchinsky A. N. Chait B. T. (2002). On the nature of the chemical noise in MALDI mass spectra. J. Am. Soc. Mass Spectrosc. 13, 129–134. 10.1016/S1044-0305(01)00336-1 11838016

[B24] Kumar R. Sripriya R. Balaji S. Senthil Kumar M. Sehgal P. K. (2011). Physical characterization of succinylated type I collagen by Raman spectra and MALDI-TOF/MS and *in vitro* evaluation for biomedical applications. J. Mol. Struct. 994, 117–124. 10.1016/j.molstruc.2011.03.005

[B25] Lanni E. J. Masyuko R. N. Driscoll C. M. Dunham S. J. B. Shrout J. D. Bohn P. W.. (2014). Correlated imaging with C60-SIMS and confocal raman microscopy: visualization of cell-scale molecular distributions in bacterial biofilms. Anal. Chem. 86, 10885–10891. 10.1021/ac5030914 25268906 PMC4221875

[B26] Lasch P. Noda I. (2017). Two-dimensional correlation spectroscopy for multimodal analysis of FT-IR, Raman, and MALDI-TOF MS hyperspectral images with Hamster brain tissue. Anal. Chem. 89, 5008–5016. 10.1021/acs.analchem.7b00332 28365985

[B27] Masyuko R. Lanni E. J. Sweedler J. V. Bohn P. W. (2013). Correlated imaging - a grand challenge in chemical analysis. Analyst 138, 1924–1939. 10.1039/c3an36416j 23431559 PMC3718397

[B28] Masyuko R. N. Lanni E. J. Driscoll C. M. Shrout J. D. Sweedler J. V. Bohn P. W. (2014). Spatial organization of *Pseudomonas aeruginosa* biofilms probed by combined matrix-assisted laser desorption ionization mass spectrometry and confocal Raman microscopy. Analyst 139, 5700–5708. 10.1039/C4AN00435C 24883432

[B29] Matousek P. Stone N. (2013). Recent advances in the development of Raman spectroscopy for deep non-invasive medical diagnosis. J. Biophot. 6, 7–19. 10.1002/jbio.201200141 23129567

[B30] Movasaghi Z. Rehman S. Rehman I. U. (2007). Raman spectroscopy of biological tissues. Appl. Spectro. Rev. 42, 493–541. 10.1080/05704920701551530

[B31] Muhamadali H. Weaver D. Subaihi A. Almasoud N. Trivedi D. K. Ellis D. I.. (2016). Chicken, beams, and Campylobacter: rapid differentiation of foodborne bacteria via vibrational spectroscopy and MALDI-mass spectrometry. Analyst 141, 111–122. 10.1039/C5AN01945A 26523729

[B32] Notingher I. Hench L. L. (2006). Raman microspectroscopy: a noninvasive tool for studies of individual living cells *in vitro*. Exp. Rev. Med. Dev. 3, 215–234. 10.1586/17434440.3.2.215 16515388

[B33] R Core Team (2017). R: A Language and Environment for Statistical Computing. Vienna: R Foundation for Statistical Computing.

[B34] Ramoji A. Neugebauer U. Bocklitz T. Foerster M. Kiehntopf M. Bauer M.. (2012). Toward a spectroscopic hemogram: Raman spectroscopic differentiation of the two most abundant leukocytes from peripheral blood. Anal. Chem. 84, 5335–5342. 10.1021/ac3007363 22721427

[B35] Ryabchykov O. Bocklitz T. Ramoji A. Neugebauer U. Foerster M. Kroegel C. (2016). Automatization of spike correction in Raman spectra of biological samples. Chemometr. Intell. Lab. Syst. 155, 1–6. 10.1016/j.chemolab.2016.03.024

[B36] Ryan C. G. Clayton E. Griffin W. L. Sie S. H. Cousens D. R. (1988). SNIP, a statistics-sensitive background treatment for the quantitative analysis of PIXE spectra in geoscience applications. Nuclear Instr. Methods Phys. Res. B 34, 396–402. 10.1016/0168-583X(88)90063-8

[B37] Schulze H. G. Turner R. F. (2014). A two-dimensionally coincident second difference cosmic ray spike removal method for the fully automated processing of Raman spectra. Appl. Spectro. 68, 185–191. 10.1366/13-07216 24480274

[B38] Singhal N. Kumar M. Virdi J. S. (2016). MALDI-TOF MS in clinical parasitology: applications, constraints and prospects. Parasitology 143, 1491–1500. 10.1017/S0031182016001189 27387025

[B39] Tolstik T. Marquardt C. Matthäus C. Bergner N. Bielecki C. Krafft C.. (2014). Discrimination and classification of liver cancer cells and proliferation states by Raman spectroscopic imaging. Analyst 139, 6036–6043. 10.1039/C4AN00211C 25271553

[B40] Urwyler S. K. Glaubitz J. (2016). Advantage of MALDI-TOF-MS over biochemical-based phenotyping for microbial identification illustrated on industrial applications. Lett. Appl. Microbiol. 62, 130–137. 10.1111/lam.12526 26582130

[B41] Van De Plas R. Yang J. Spraggins J. Caprioli R. M. (2015). Image fusion of mass spectrometry and microscopy: a multimodality paradigm for molecular tissue mapping. Nat. Methods 12:366. 10.1038/nmeth.3296 25707028 PMC4382398

[B42] Verwer P. E. Van Leeuwen W. B. Girard V. Monnin V. Van Belkum A. Staab J. F.. (2014). Discrimination of Aspergillus lentulus from *Aspergillus fumigatus* by Raman spectroscopy and MALDI-TOF MS. Eur. J. Clin. Microbiol. Infect. Dis. 33, 245–251. 10.1007/s10096-013-1951-4 24030717

[B43] Wagner M. (2009). Single-cell ecophysiology of microbes as revealed by Raman microspectroscopy or secondary ion mass spectrometry imaging. Ann. Rev. Microbiol. 63, 411–429. 10.1146/annurev.micro.091208.073233 19514853

[B44] Zhang L. Henson M. J. (2007). A practical algorithm to remove cosmic spikes in Raman imaging data for pharmaceutical applications. Appl. Spectro. 61, 1015–1020. 10.1366/000370207781745847 17910800

