# Supplementary material for: Fusion of MALDI Spectrometric Imaging and Raman Spectroscopic Data for the Analysis of Biological Samples
Source: Front Chem. 2018 Jul 16;6:257. doi: 10.3389/fchem.2018.00257 (PMC6055053; doi:10.3389/fchem.2018.00257)
Supplement: Supplementary Image 1 — The plots from Figure 6 provided in vector format. [file Image_1.PDF]

Fusion of MALDI Spectrometric Imaging and Raman Spectroscopic Data for the Analysis of Biological Samples

Supplementary material

Oleg Ryabchykov, Juergen Popp, Thomas Bocklitz

Leibniz Institute of Photonic Technology, Jena, Germany
Institute of Physical Chemistry and Abbe Center of Photonics,
Friedrich Schiller University Jena, Jena, Germany

Correspondence: Thomas Bocklitz, Institute of Physical Chemistry and Abbe Center of Photonics,
Friedrich−Schiller−University, Helmholtzweg 4, 07743 Jena, Germany.
thomas.bocklitz@uni−jena.de

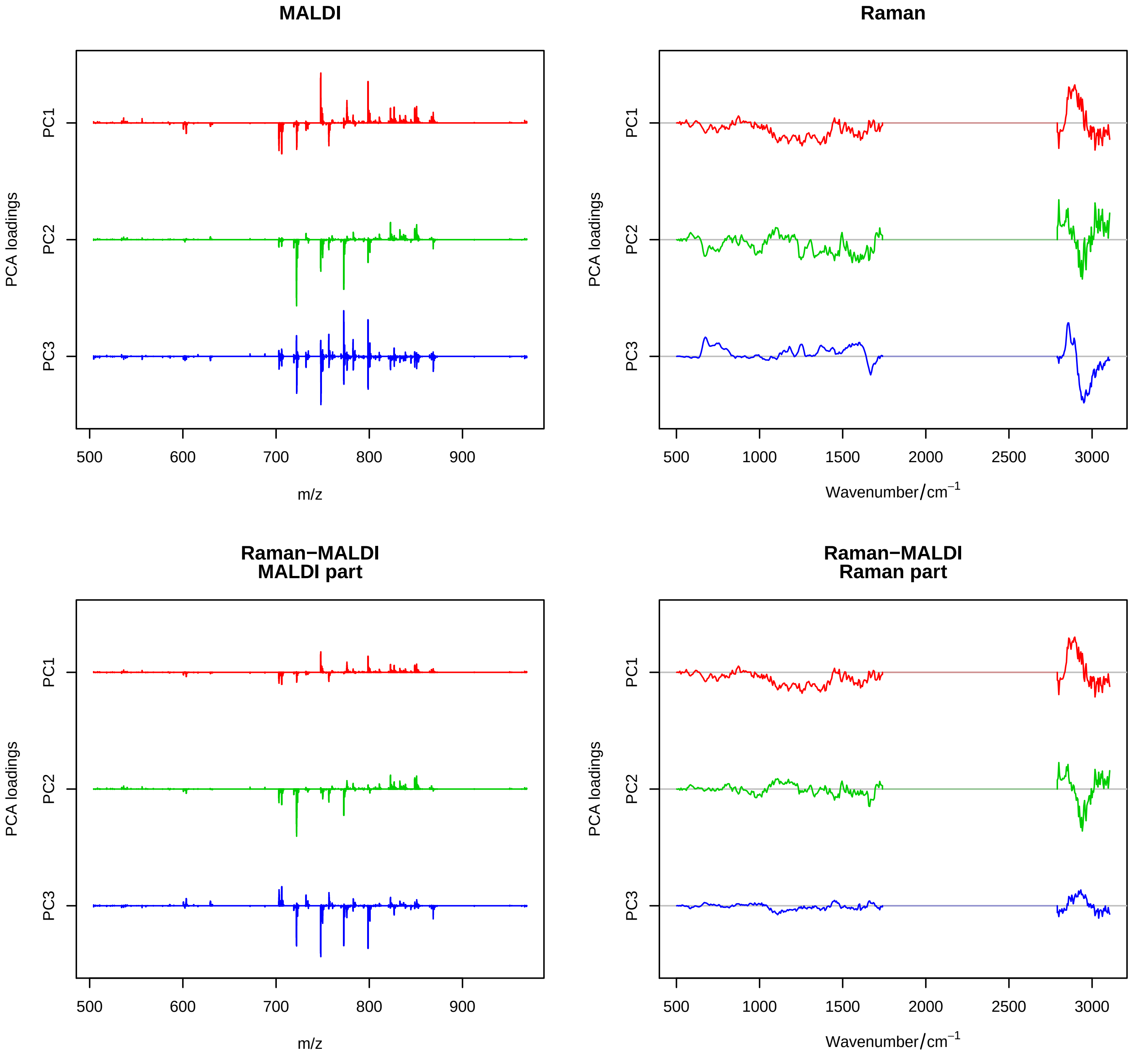

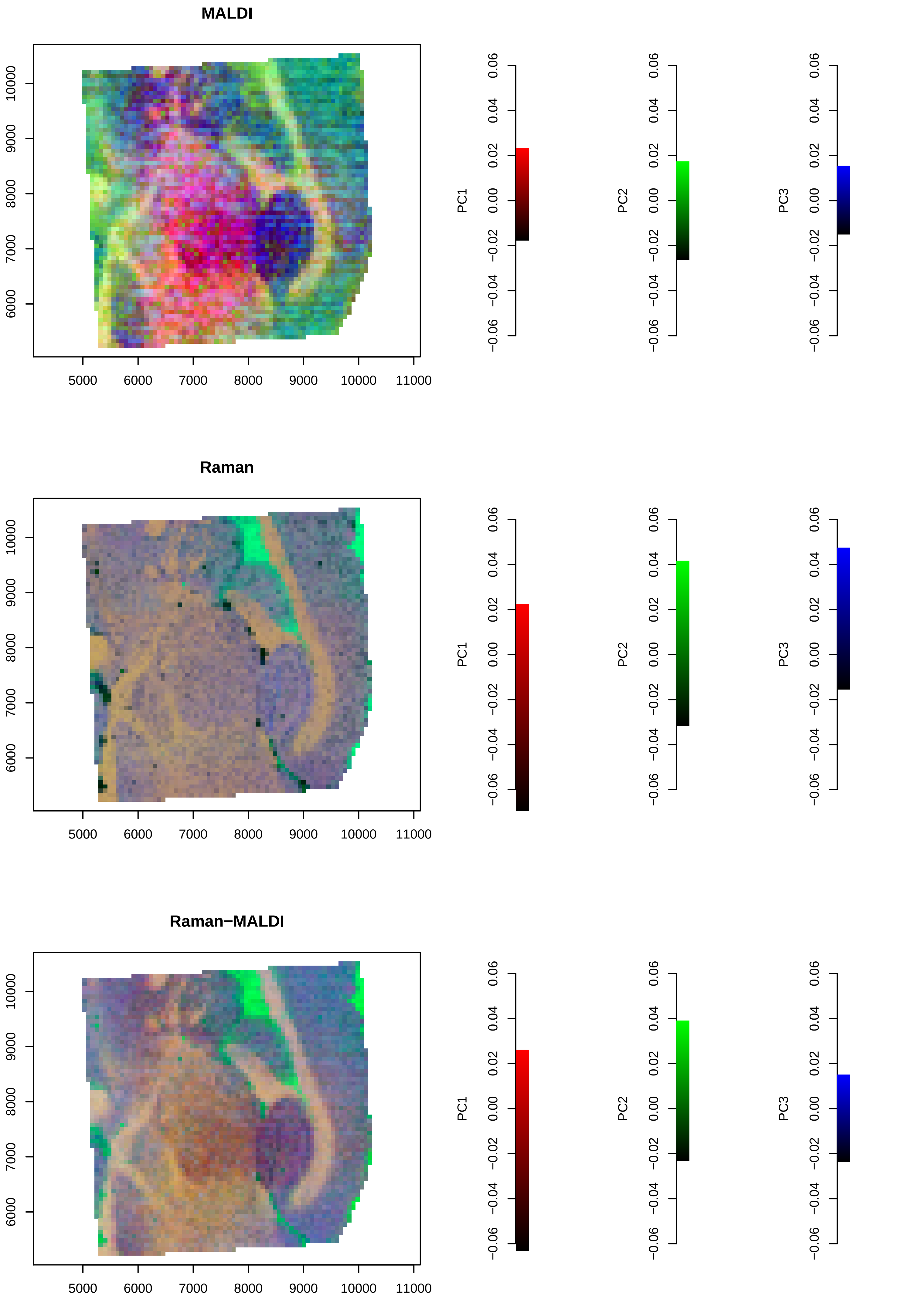
MALDI
10000
9000
8000
7000
6000
5000
6000
7000
8000
9000
10000
11000
PC1
PC2
PC3
0.06
0.04
0.02
0.00
−0.02
−0.04
−0.06
Raman
Raman−MALDI
